# GAS5 Regulates RECK Expression and Inhibits Invasion Potential of HCC Cells by Sponging miR-135b

**DOI:** 10.1155/2019/2973289

**Published:** 2019-01-13

**Authors:** Liang Yang, Jianshuai Jiang

**Affiliations:** Department of Hepatobiliary and Pancreatic Surgery, Ningbo First Hospital, Zhejiang, Ningbo 315010, China

## Abstract

**Objectives:**

Long noncoding RNA (LncRNA) growth arrest-specific 5 (GAS5) has been characterized as a tumor suppressor in numerous kinds of human cancers. Its anticancer function in hepatocellular carcinoma (HCC) includes repression of cell proliferation and metastasis, leaving the internal mechanisms unclear. In this study, we intended to examine the anti-invasion effects of GAS5 on HCC and explore the downstream regulatory mechanisms.

**Methods:**

Expression of GAS5 and microRNA-135b (miR-135b) was analyzed by qRT-PCR in paired HCC tissue samples. Their correlation with HCC patients' survival was determined. Transwell assays were done to evaluate* in vitro* invasion ability. Targeting of GAS5 and RECK by miR-135b was confirmed by qRT-PCR, western blot, and luciferase reporter assays.

**Results:**

Decreased GAS5 and increased miR-135b in HCC inversely correlate with each other and both correlate with poor prognosis of HCC patients. Functionally, GAS5 suppresses while miR-135b promotes HCC cell invasion capacities* in vitro*. Mechanistically, GAS5 is a target of miR-135b. Furthermore, GAS5 positively regulates expression of RECK, also a target of miR-135b, which further inhibits MMP-2 expression and contributes to invasion repression.

**Conclusion:**

GAS5 acted as a tumor suppressor in HCC invasion in a competing endogenous RNA manner. Our findings indicate that GAS5 is a promising therapeutic target for HCC treatment.

## 1. Introduction

According to the latest statistics, hepatocellular carcinoma (HCC) is the sixth common malignancy over the world, and the main cause of mortality for patients with cirrhosis [1, 2]. Accounting for over 85% of all primary liver cancers, HCC is associated with poor prognosis and becomes incurable because of occurrence of intrahepatic and extrahepatic metastasis [3, 4]. Carcinogenesis of HCC is known to be a sequential multistep process consisting of various genetic and epigenetic alterations [5]. Current HCC therapy options include surgery, biotherapy, and chemotherapy. Nevertheless, it is the dimness of HCC pathophysiological mechanisms that largely limits treatment effect of this disease. Keeping on investigating more on its pathological mechanism driving HCC progression is of great importance for drug development.

In the past few decades, studies on HCC tumorigenesis mostly focused on protein coding genes and their functions. Surprisingly, recent progress on noncoding RNAs refreshes our understanding of cancer development [6]. Apart from microRNAs (miRNAs), long noncoding RNAs (lncRNAs) also exhibit important regulatory roles in cellular functions and carcinogenesis [7]. LncRNAs are defined by longer than 200 nucleotides in length and short of protein coding potentials [8]. Increasing evidence verify that dysregulation of lncRNAs are associated with multiple human cancers, several of which can be recognized as prognosis biomarkers with gratifying therapy effects in cancers [9–11].

GAS5 (growth arrest-specific transcript 5) was firstly identified using a functional cDNA library screen, which is mainly expressed in growth-arrested cells [12]. GAS5 gene contains 12 exons, whose open reading frame is quite short and considered to be unable to encode protein. Various literatures identify GAS5 as a tumor suppressing lncRNA: for instance, in non-small cell lung cancer, GAS5 is involved in cell proliferation, apoptosis, and migration [13]. In HCC, several reports have demonstrated that decreased GAS5 expression is correlated with poor prognosis and enhanced proliferation, migration, and invasion [14–16]. However, the mechanism mediating its anticancer roles remains unclear and deserves further investigation. Moreover, recent studies prove that lncRNAs can act as competing endogenous RNAs (ceRNAs), in other words, as molecular sponges in regulating the concentration and biological effects of miRNAs [17, 18]. However, GAS5/miRNA trans-regulatory network remains unclear.

RECK (reversion-inducing cysteine-rich protein with Kazal motifs) has important implications in cancer biology, especially in regulation matrix metalloproteinases (MMPs) [19, 20]. It is decreased in cells undergoing malignant transformation and also negatively contributes to cancer metastasis [19–21]. Studies also reveal that RECK has inhibitory effects on HCC invasion and its silencing is associated with poor survival of HCC patients [22]. However, the correlation between GAS5 and RECK remains unknown.

In this study, initial bioinformatics analysis showed a potential interaction between GAS5 and miR-135b. Therefore, we speculated that GAS5 might function as a miR-135b sponge in HCC. Hence, we detected the expression of GAS5 and miR-135b in HCC tissues and cell lines, determined the relationship between GAS5 and miR-135b, investigated their roles in HCC cell invasion, and finally validated the impact of GAS5 in regulation of RECK by sponging miR-135b.

## 2. Materials and Methods

### 2.1. Patients and Tissues

A total of 50 patients at the Ningbo First Hospital from January 2014 to December 2016 were analyzed in this study. The study was approved by the Ethics Committee of Ningbo First Hospital. Specimen collection was approved by the Ethics Committee of Ningbo First Hospital and written informed consent was obtained from all patients. All patients involved in our research were underwent hepatic surgical resection without postoperative systemic chemotherapy from January 2014 to December 2016. These selected cases have been examined by experienced pathologist's histological examination of H&E stained biopsy sections. The clinical data of all 50 patients were displayed in Supplementary Table I. The 50 HCC tissues samples and 50 paired adjacent noncancerous liver tissue samples were frozen in liquid nitrogen immediately after surgery, followed by stored at –80°C before used.

### 2.2. Cell Culture

The HCC cell lines Huh-7, HB611, BEL-7404, Hep3B, HepG2, and human normal hepatocytes QSG-7701 were purchased from the Cell Bank of the Chinese Academy of Sciences (Shanghai, China). All cell lines were kept in our laboratory in liquid nitrogen for long-stem storage as per the manufacturer's instructions. The cells were cultured in RPMI-1640 medium (Gibco, Life Technology, USA) containing 10% fetal bovine serum (FBS, Gibco, Life Technology, USA) in a humidified 37°C incubator containing 5% CO_2_.

### 2.3. Lentivirus and Infections

Lentiviruses expressing miR-135b (miR-135b-LV), its scrambled control lentivirus (miR-C-LV), GAS5 (GAS5-LV), RECK (RECK-LV), and the blank control (Con-LV) were all purchased from Genepharma (Shanghai, China). Huh-7 and HepG2 cells were infected with lentivirus at a MOI of 20 using 10*μ*g/ml polybrene (Sigma, St. Louis, MO, USA).

### 2.4. miRNA Inhibitor, siRNAs, and Transfections

MiR-135b inhibitor, GAS5 siRNAs, RECK siRNAs, and MMP-2 siRNAs were purchased from Genepharma (Shanghai, China). Huh-7 and HepG2 cells were transfected using Lipofectamine®RNAiMAX as per the manufacturer's instructions and collected 72h later for other assays.

### 2.5. Plasmid Construction

The wild-type or mutant sequence of GAS5 was cloned into pmirGLO vector (Promega, Madison, WI, USA). The wild-type or mutant sequence of 3'-UTR region of RECK promoter was cloned into pmirGLO vector (Promega, Madison, WI, USA). The wild-type sequence of MMP-2 was cloned into pcDNA3.1 vector (Life Technology, USA). Huh-7 and HepG2 cells were transfected using Lipofectamine®2000 as per the manufacturer's instructions.

### 2.6. Quantitative Real-Time PCR (qRT-PCR)

Total RNAs were extracted from tissues or cells by using TRIzol reagent (Life Technology) according to the manufacturer's instructions. qRT-PCR was performed to determine the expression levels of lncRNA GAS5, miR-135b, and other genes. 1*μ*g of RNA was reverse transcribed to detect miR-135b and U6B expression level using specific primer kit (Ribobio, Guangzhou, China). 2*μ*g of RNA was reverse transcribed to detect lncRNA, *β*-actin, RECK, and MMP-2 expression level using general reverse transcription kit (Promega, Madison, WI, USA). These products were subjected to quantitative PCR to detect lncRNA, microRNA and gene expressions using the SYBR Green qPCR Master Mix (TOYOBO, Osaka, Japan). U6B and *β*-actin were served as internal controls for microRNA and lncRNA, respectively.

### 2.7. Western Blot

Huh-7 and HepG2 cells were lysed by RIPA buffer (Beyotime, Beijing, China) with 1mM PMSF (Beyotime, Beijing, China). Total proteins were separated by SDS-PAGE (sodium dodecyl sulfate-polyacrylamide gel electrophoresis) and transferred to a NC membrane (Beyotime, Beijing, China), followed by blocking with skim milk and probing with primary antibodies against RECK, MMP-2, and *β*-actin (Cell Signaling Technology, USA). Immune complexes were detected by enhanced chemiluminescence (Pierce, Rockford, IL, USA).

### 2.8. Cell Invasion Assays

For cell invasion assay, 2×10^5^ cells were placed in the Matrigel (BD Biosciences, Billerica, MA, USA)-coated upper chamber of each insert. After incubation at 37°C for 4 hours, cells remaining in the upper chambers were removed with cotton swabs, while cells adhering to the lower membrane were stained with 0.25% crystal violet in PBS. Cells that had invaded into the lower membrane were photographed and counted under an inverted microscope (Olympus, Tokyo, Japan).

### 2.9. Dual Luciferase Reporter Assay

Huh-7 and HepG2 cells were seeded in 24-well plates at a density of 2×10^5^ cells per well 24h before transfection. Cells were transfected with a mixture of 0.05*μ*g firefly luciferase reporter and 0.01*μ*g Renilla luciferase reporter (pRL-CMV, Promega, Madison, WI, USA). 12h later, cells were infected with miR-135b/GAS5 overexpressing or control lentivirus. 72h later, cells were harvested and firefly and Renilla luciferase activities were measured using the dual luciferase reporter assay (Promega, Madison, WI, USA).

### 2.10. Target Prediction

The interactions of lncRNA GAS5 and miRNA has-miR-135b-5p were predicted by the online bioinformatics algorithms: starBase v2.0 (http://starbase.sysu.edu.cn/index.php). The target genes of has-miR-135b-5p were predicted by the online bioinformatics algorithms: TargetScan Release 3.1 (http://www.targetscan.org/mamm_31/).

### 2.11. Statistical Analysis

All statistical analyses were performed using Graphpad software v6.0. The difference between expressions in paired samples was calculated using paired Student's t-test. The difference between groups was calculated using grouped Student's t-test. Survival curves were analyzed using Kaplan-Meier and log-rank analyses. The correlation between GAS5 and miR-135b expression in paired samples was calculated by Spearman's rank correlation. Data represent the mean±SD from three independent experiments. A P value of <0.05 was considered different significantly.

## 3. Results

### 3.1. Inverse Correlation between Expression of GAS5 and miR-135b

**Figure 1:**
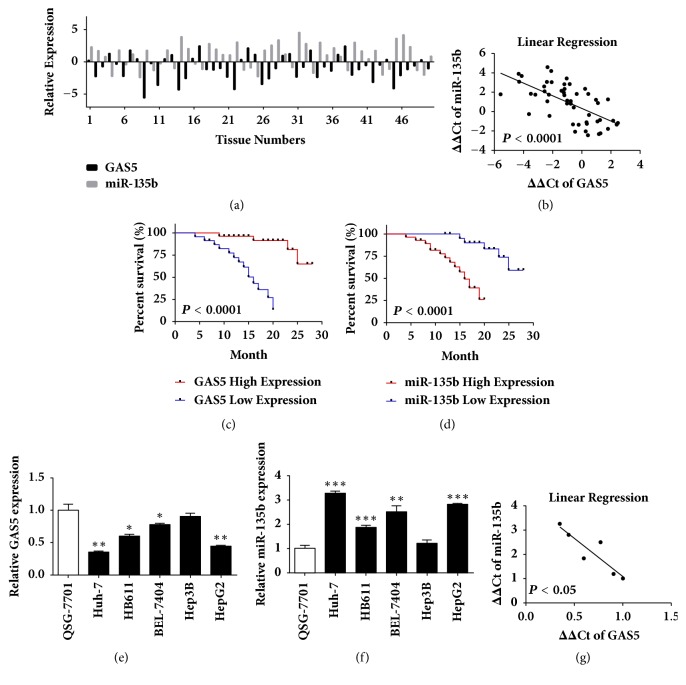
**Inverse correlation between expression of GAS5 and miR-135b in paired HCC samples and HCC cell lines.** (a) GAS5 and miR-135b expression was detected by qRT-PCR relative to the internal control *β*-actin or U6B in 50 HCC tissue samples and 50 paired adjacent noncancerous liver tissue samples. (b) Correlation between GAS5 and miR-135b expressions in paired HCC samples (n=50). *β*-actin and U6B were served as internal controls, respectively. Statistical analysis was performed by Pearson's correlation analysis. (c) Low expression of GAS5 (ΔΔCt<–1) associated with poor prognosis in HCC. Kaplan-Meier analysis of survival months after surgery was performed according to GAS5 expression level. (d) High expression of miR-135b (ΔΔCt>1) associated with poor prognosis in HCC. Kaplan-Meier analysis of survival months after surgery was performed according to miR-135b expression level. (e) The expression of GAS5 was lower in HCC cell lines compared with QSG-7701 normal hepatocytes assessed by qRT-PCR assay. *β*-actin was used as an internal control. (f) The expression of miR-135b was higher in HCC cell lines compared with QSG-7701 normal hepatocytes assessed by qRT-PCR assay. U6B was used as an internal control. (g) Correlation between GAS5 and miR-135b expressions in HCC cell lines. *β*-actin or U6B was served as an internal control, respectively. Statistical analysis was performed by Pearson's correlation analysis. Data represent the mean±SD from three independent experiments. *∗*: P<0.05, *∗∗*: P<0.01, and *∗∗∗*: P<0.001.

Numerous studies have demonstrated that lncRNA GAS5 is downregulated in various types of human cancers, including HCC [14–16]. MiR-135b, known as an overexpressing and oncogenic miRNA in human cancers, is associated with tumorigenesis and invasiveness [23–25]. To determine the expression correlation between the two ncRNAs, their levels were analyzed by qRT-PCR in 50 HCC tissues and adjacent nontumor tissues. The results showed that GAS5 expression was significantly downregulated while miR-135b was upregulated in HCC tissues compared with paired nontumor tissues (Figure 1(a)). Correlation analysis showed an inverse correlation between expression levels of GAS5 and miR-135b (Figure 1(b)), indicating a negative regulatory mechanism between the two ncRNAs. Furthermore, the association between GAS5 or miR-135b expression and overall survival of HCC patients was then evaluated by Kaplan-Meier analysis. Higher GAS5 predicted better prognosis displaying longer survival time (Figure 1(c)). However, higher miR-135b predicted worse prognosis (Figure 1(d)). We further detected the expression levels of GAS5 and miR-135b in HCC cell lines. Finely consistent with the tissue expression patterns, we found that GAS5 was downregulated while miR-135b was upregulated in several HCC cell lines, in comparison with that in the human normal hepatocytes QSG-7701, respectively (Figures 1(e) and 1(f)). Correlation analysis also showed an inverse correlation between expression levels of GAS5 and miR-135b in the tested cell lines (Figure 1(g)). Therefore, our results confirmed the downregulation of GAS5 and upregulation of miR-135b and their prognostic significance in HCC, implying their negative correlation with each other.

### 3.2. GAS5 Represses While miR-135b Promotes HCC Cell Migration and Invasion* In Vitro*

**Figure 2:**
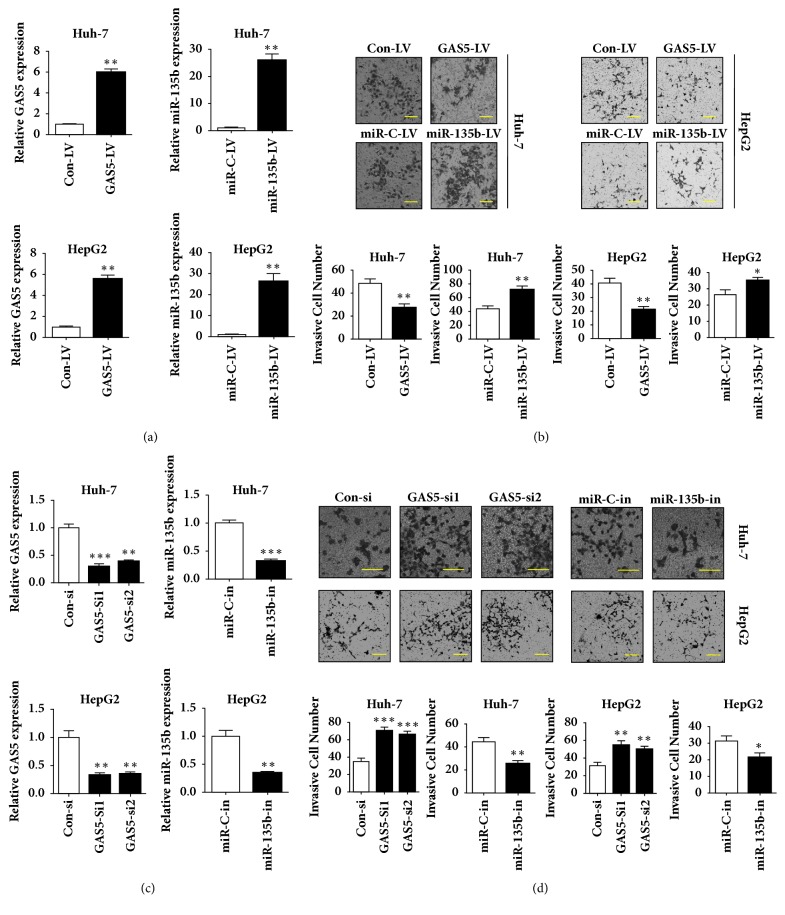
**GAS5 represses while miR-135b promotes HCC cell invasion* in vitro*.** (a) Huh-7 and HepG2 cells were infected by lentivirus (LV) expressing GAS5 or miR-135b. 72h later, the expressions of GAS5 or miR-135b were detected by qRT-PCR. (b) Cell invasion assay was performed to investigate the invasion ability of GAS5 or miR-135b overexpression using stable cells as described in (a). (c) Huh-7 and HepG2 cells were transfected with GAS5 siRNAs or miR-135b inhibitor. 72h later, the expressions of GAS5 or miR-135b were detected by qRT-PCR. (d) Cell invasion assay was performed to investigate the invasion ability of GAS5 or miR-135b knockdown using transfected cells as described in (c). Data represent the mean±SD from three independent experiments. *∗*: P<0.05, *∗∗*: P<0.01, and *∗∗∗*: P<0.001. Scale bar: 50*μ*m.

To elucidate the function of GAS5 and miR-135b in HCC invasion* in vitro*, we firstly established four types of stable cell lines by infecting Huh-7 and HepG2 cells with indicated lentivirus (lentivirus expressing control, GAS5, miR-C, or miR-135b). qRT-PCR confirmed the overexpression of GAS5 or miR-135b in Huh-7 and HepG2 cells (Figure 2(a)). Transwell assays demonstrated that GAS5 repressed while miR-135b promoted HCC cell invasion (Figure 2(b)). We then used two independent siRNAs toward GAS5 to inhibit GAS5 expression and used the inhibitor for miR-135b to inhibit miR-135b expression (Figure 2(c)). Also,* in vitro* invasion capacity analysis showed that inhibition of GAS5 promoted while inhibition of miR-135b repressed HCC cell invasion (Figure 2(d)). Thus, through gain/loss-of-function studies, we concluded that GAS5 inhibited while miR-135b enhanced HCC invasion* in vitro*.

### 3.3. GAS5 Regulates miR-135b Expression and Is a Target of miR-135b

**Figure 3:**
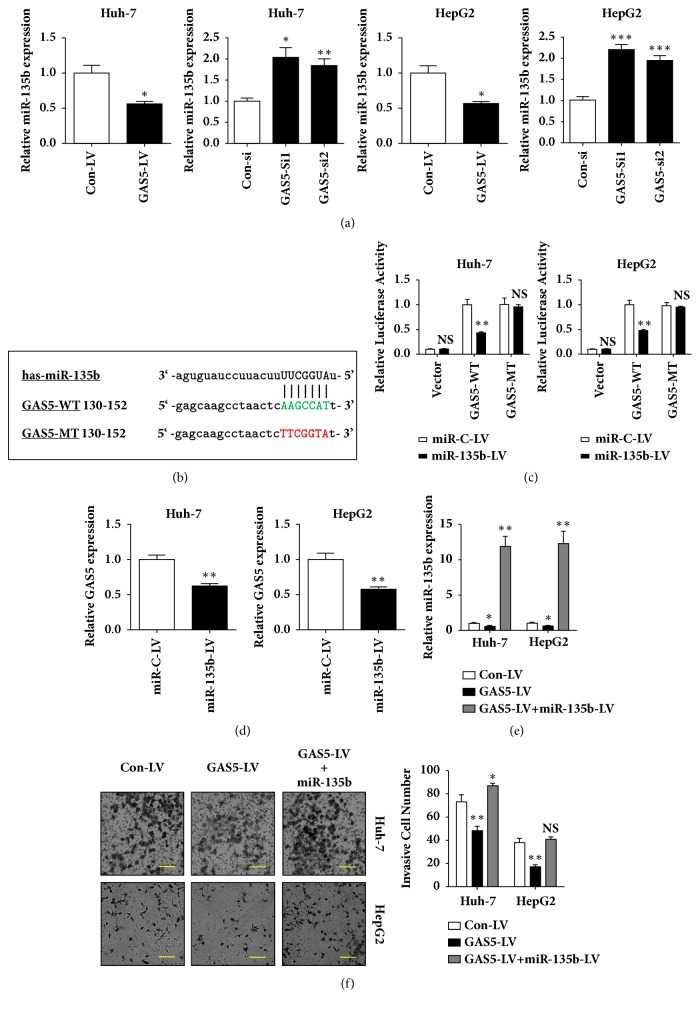
**GAS5 regulates miR-135b expression and is a direct target of miR-135b.** (a) Huh-7 and HepG2 cells were infected by lentivirus (LV) expressing GAS5. Huh-7 and HepG2 cells were transfected with GAS5 siRNAs. 72h later, the expression of miR-135b was detected by qRT-PCR. (b) Putative miR-135b binding sites in GAS5 lncRNA sequence by starBase v2.0 online software. Wild-type (WT) binding sequences were highlighted in green, while mutant (MT) binding sequences were highlighted in red. (c) Huh-7 and HepG2 cells were cotransfected with wild-type or mutant GAS5 reporters and miR-135b overexpressing or control lentivirus. 72h later, cells were harvested and firefly luciferase activity was normalized to Renilla luciferase activity. (d) Huh-7 and HepG2 cells were infected by lentivirus (LV) expressing miR-135b. 72h later, the expression of GAS5 was detected by qRT-PCR. (e) Huh-7 and HepG2 cells were infected by lentivirus (LV) expressing GAS5, without or with miR-135b-LV. 72h later, the expression of miR-135b was detected by qRT-PCR. (f) Cell invasion assay was performed to investigate the invasion ability of miR-135b in GAS5-overexpressing cells as described in (e). Data represent the mean±SD from three independent experiments. *∗*: P<0.05, *∗∗*: P<0.01, and NS: no significance. Scale bar: 50*μ*m.

From the above results, we found an inverse correlation between GAS5 and miR-135b. Given that lncRNAs could function as ceRNAs, namely as molecular sponges to control the level and roles of corresponding miRNAs, we then tested whether miR-135b interacted with and targeted GAS5. Bioinformatics prediction by starBase v2.0 found about 20 miRNA binding sites within GAS5 sequence. Among these miRNAs, miR-135b attracted our attention since we had proved an inverse correlation between GAS5 and miR-135b expression levels in HCC tissues and cell lines. In addition, we found that miR-135b expression was decreased in GAS5 overexpressing Huh-7 and HepG2 cells, whereas GAS5 siRNA treatment increased miR-135b level (Figure 3(a)). To explore whether GAS5 is a target of miR-135b as predicted by starBase v2.0, we constructed two luciferase reporters of GAS5 (GAS5-WT and GAS5-MT; Figure 3(b)). Overexpression of miR-135b mediated by lentivirus caused reduced luciferase activity of GAS5-WT; however, overexpression of miR-135b failed to suppress the luciferase activity of GAS5-MT, in which miR-135b binding sites were mutated (Figure 3(c)). Moreover, GAS5 expression was significantly decreased in miR-135b-overexpressed Huh-7 and HepG2 cells (Figure 3(d)). These results suggested that miR-135b could directly interact with GAS5 and GAS5 is a target of miR-135b in HCC cells. More importantly, restoration of miR-135b in GAS5-overexpressing cells abolished the invasion-suppressing effect of GAS5 (Figures 3(e) and 3(f)).

### 3.4. GAS5 Regulates RECK Expression via Sponging miR-135b

**Figure 4:**
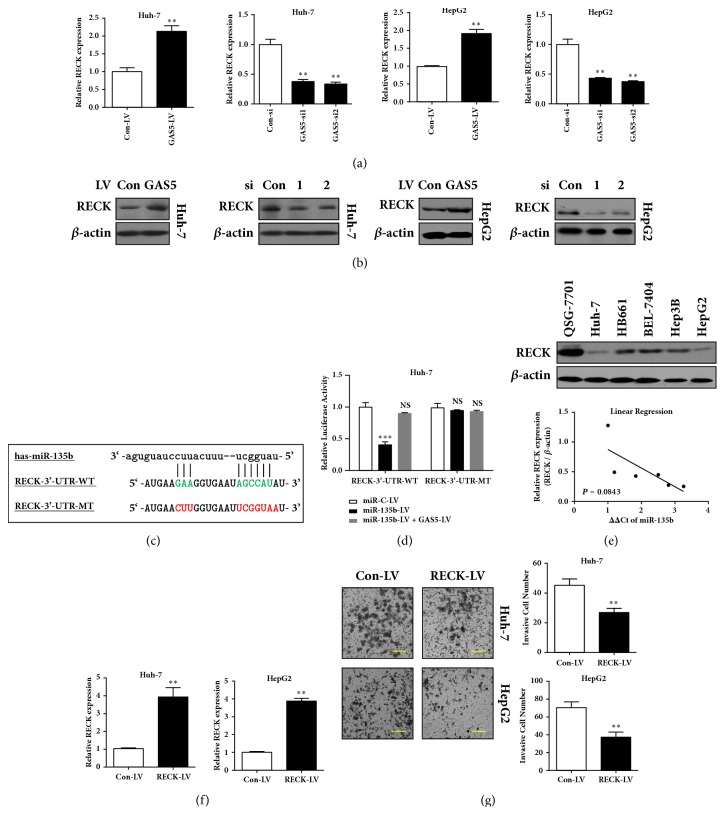
**GAS5 regulates RECK expression via sponging miR-135b.** (a) Huh-7 and HepG2 cells were infected by lentivirus (LV) expressing GAS5. Huh-7 and HepG2 cells were transfected with GAS5 siRNAs. 72h later, the expression of RECK was detected by qRT-PCR. (b) Huh-7 and HepG2 cells were infected by lentivirus (LV) expressing GAS5. Huh-7 and HepG2 cells were transfected with GAS5 siRNAs. 72h later, the expression of RECK was detected by western blot. (c) Putative miR-135b binding sites in the RECK 3'-UTR (3'-untranslated regio) predicted by TargetScan Release 3.1 online software. Wild-type (WT) binding sequences were highlighted in green. Mutant (MT) binding sequences were highlighted in red. (d) Huh-7 cells were cotransfected with wild-type or mutant RECK 3'-UTR reporters with miR-135b/GAS5 overexpressing or control lentivirus. 72h later, cells were harvested and firefly luciferase activity was normalized to Renilla luciferase activity. (e) The expression of RECK was lower in HCC cell lines compared with QSG-7701 normal hepatocytes assessed by western blot. *β*-actin was used as an internal control. The bottom panels showed the correlation between miR-135b and RECK expression in HCC cell lines. U6B or *β*-actin was served as an internal control, respectively. Statistical analysis was performed by Pearson's correlation analysis. (f) Huh-7 and HepG2 cells were infected by lentivirus (LV) expressing RECK. 72h later, the expression of RECK was detected by qRT-PCR. (g) Cell invasion assay was performed to investigate the invasion ability of RECK overexpression using stable cells as described in (f). Data represent the mean±SD from three independent experiments. *∗∗*: P<0.01, *∗∗∗*: P<0.001, and NS: no significance. Scale bar: 50*μ*m.

Among the numerous predicted targets of miR-135b by bioinformatics analysis (TargetScan Release v3.1), we centered on RECK, one validated target of miR-135b in HCC cells which is associated with invasion and metastasis of HCC cells [26]. We then intended to detect whether GAS5 modulated the expression of RECK. As shown in Figure 4(a), RECK mRNA was increased in GAS5 overexpressing cells and decreased by GAS5 siRNA treatment in Huh-7 and HepG2 cells. Western blot showed the consistent alteration of RECK protein level by GAS5 overexpression or knockdown (Figure 4(b)). Moreover, luciferase reporter assay found that overexpression of miR-135b mediated by lentivirus inhibited the luciferase activity of wild-type RECK 3'-UTR (RECK-3'-UTR-WT), whereas coinfection of GAS5 and miR-135b overexpressing lentiviruses repressed the miR-135b induced decrease of wild-type RECK 3'-UTR activity (Figures 4(c) and 4(d)). Moreover, we found that RECK was downregulated in a panel of HCC cell lines compared with nontumor hepatocytes QSG-7701 (Figure 4(e)), and nearly inversely correlated with miR-135b in these cell lines (Figure 4(e),* P*=0.0843), further implying the targeting of RECK by miR-135b. Besides, overexpression of RECK suppressed the invasion of HCC cells (Figures 4(f) and 4(g)). Therefore, these data suggested that GAS5 could regulate RECK expression by sponging miR-135b, and RECK was an inhibitor of HCC invasion.

### 3.5. MMP-2 Inhibition Contributes to Invasion Repression Caused by GAS5

**Figure 5:**
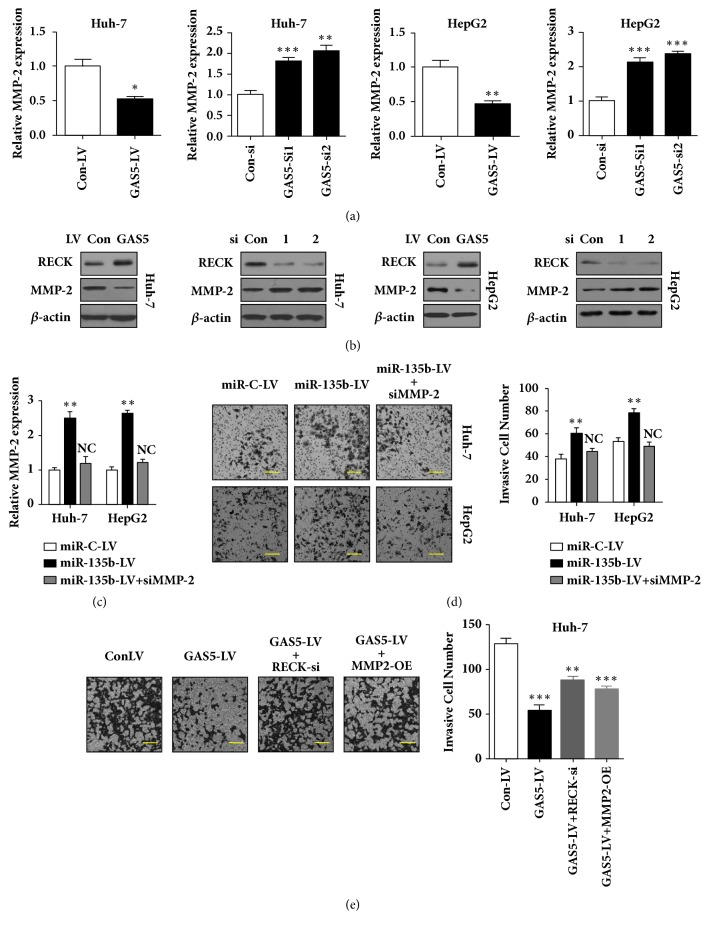
**MMP-2 inhibition contributes to invasion repression caused by GAS5.** (a) Huh-7 and HepG2 cells were infected by lentivirus (LV) expressing GAS5. Huh-7 and HepG2 cells were transfected with GAS5 siRNAs. 72h later, the expression of MMP-2 was detected by qRT-PCR. (b) Huh-7 and HepG2 cells were infected by lentivirus (LV) expressing GAS5. Huh-7 and HepG2 cells were transfected with GAS5 siRNAs. 72h later, the expressions of RECK and MMP-2 was detected by western blot. (c) Huh-7 and HepG2 cells were infected by lentivirus (LV) expressing miR-135b, without or with MMP-2 siRNAs. 72h later, the expression of MMP-2 was detected by qRT-PCR. (d) Cell invasion assay was performed to investigate the invasion ability of MMP-2 knockdown in miR-135b-overexpressing cells as described in (c). (e) Cell invasion assay was performed to investigate the function of RECK and MMP-2 in invasion repression caused by GAS5. Data represent the mean±SD from three independent experiments. *∗*: P<0.05, *∗∗*: P<0.01, and *∗∗∗*: P<0.001. Scale bar: 50*μ*m.

Previous studies indicate a linkage between RECK and ECM destruction by inhibiting MMP-2 [26, 27]. From the above findings, we found that GAS5 positively regulated RECK expression. We also investigated whether GAS5 regulates MMP-2 expression and activity. qRT-PCR and western blot assays indicated that MMP-2 expression was decreased by GAS5 overexpression or increased by GAS5 siRNA treatment in Huh-7 and HepG2 cells (Figures 5(a) and 5(b)). In addition, knockdown of MMP-2 in miR-135b-overexpressing cells abolished the invasion-promoting activity of miR-135b (Figures 5(c) and 5(d)). Furthermore, RECK knockdown or MMP-2 overexpression in GAS5 overexpressing Huh-7 cells largely reverses the invasion repression caused by GAS5 as gauged by transwell assay (Figure 5(e)). These results indicated that GAS5 inhibited both expression and activity of MMP-2 by inducing RECK expression, thereby repressing HCC cell invasion.

## 4. Discussion

It is widely recognized that lncRNAs are linked with cancer development and involved in various biological processes. Intensive studying of the detailed mechanisms of actions of oncogenic or tumor suppressing lncRNAs certainly provides more insights into the pathogenetic process and contributes to novel therapy for human cancers. Notably, more and more studies prove that lncRNAs can act as ceRNAs or molecular sponges in regulating the concentration and biological effects of miRNAs when the expression of lncRNA and miRNA shows an inverse correlation [17, 18]. GAS5 is a nonprotein coding gene and mainly expressed in growth-arrested cells [12]. Multiple studies have proved that GAS5 is dysregulated in human cancers and functions as a tumor suppressing lncRNA [13–16]. However, little is known about its role and mechanism in HCC.

In the present study, we firstly found that GAS5 was decreased in HCC tissues and cell lines, and exhibited an inverse correlation with increased miR-135b. Both decreased GAS5 and increased miR-135b were correlated with poor prognosis of HCC patients. In cellular experiments, we found that GAS5 suppresses while miR-135b promotes HCC cell invasion capacities. Moreover, we found that GAS5 could regulate miR-135b expression and is a target of miR-135b. Restoration of miR-135b in GAS5-overexpressing cells abolished the invasion-suppressing effect of GAS5. Besides, GAS5 positively regulates expression of RECK, a known target of miR-135b, which further inhibit MMP-2 expression and contributes to invasion repression in HCC cells.

MiR-135b has been characterized to be upregulated in various types of cancers, including HCC [26], and it has been identified as a crucial downstream effector in oncogenic pathways and a potential therapeutic target for colon cancer [28]. Herein in HCC, we found that miR-135b was increased in HCC tissues compared to adjacent nontumor tissues, and its upregulation predicted shorter overall survival of HCC patients. We further found that miR-135b promoted HCC cell invasion* in vitro* and identified at least two targets of miR-135b, GAS5 and RECK. The targeting of GAS5 by miR-135b was strengthened by the negative correlation between expression levels of GAS5 and miR-135b. Targeting of RECK has been proven by a previous report [26], we confirmed this by performing luciferase reporter assays in HCC cells. Our findings regarding miR-135b kept consistency with former studies [26, 28].

RECK is an antimetastatic gene cloned from NIH3T3 cells encoding an extracellular protein and is a tumor suppressor through inhibiting MMPs thereby suppressing tumor invasion [29]. RECK is known to be downregulated in human cancers resulting from transcription or epigenetic changes [30–32]. Besides targeting by miR-135b, we also showed that GAS5 positively regulated RECK expression, therefore inhibited MMP-2 activity. Luciferase reporter assay showed that GAS5 repressed the miR-135b induced decrease of wild-type RECK 3'-UTR activity. Collectively, GAS5 positively regulated RECK expression by sponging miR-135b. Importantly, we further found that MMP-2 inhibition contributes to invasion repression caused by GAS5. Therefore, we concluded that GAS5 may inhibit cell invasion of HCC cells through inducing RECK expression via sponging miR-135b.

However, we also remind ourselves that ceRNA activity of GAS5 may enable it to sponge many miRNAs, and one miRNA can simultaneously target multiple genes. The cellular phenotypes we observed are likely caused simultaneous targeting of multiple targets by GAS5 or miR-135b in HCC. Discovery of these ceRNAs will benefit the treatment of HCC.

In summary, we identified a tumor suppressing role of GAS5 in HCC invasion by positively regulating RECK expression in a ceRNA manner. Our findings might offer a potential prognosis biomarker and therapeutic target for HCC treatment.

## Data Availability

The data used to support the findings of this study are available from the corresponding author upon request.

## Supplementary Materials

Supplementary Materials Supplementary Table I: the clinicopathological characteristics of the donor patients in this study.

## References

[1] Fidler M. M., Bray F., Soerjomataram I. (2018). The global cancer burden and human development: A review. *Scandinavian Journal of Public Health*.

[2] Spârchez Z., Mocan T. (2017). Hepatocellular carcinoma occurrence and recurrence after antiviral treatment in HCV-related cirrhosis. Are outcomes different after direct antiviral agents? A review. *Journal of Gastrointestinal and Liver Diseases*.

[3] El-Serag H. B., Rudolph K. L. (2007). Hepatocellular carcinoma: epidemiology and molecular carcinogenesis. *Gastroenterology*.

[4] Dutta R., Mahato R. I. (2017). Recent advances in hepatocellular carcinoma therapy. *Pharmacology & Therapeutics*.

[5] Nishida N., Kudo M. (2016). Clinical significance of epigenetic alterations in human hepatocellular carcinoma and its association with genetic mutations. *Digestive Diseases*.

[6] Xue M., Zhuo Y., Shan B. (2017). MicroRNAs, long noncoding RNAs, and their functions in human disease. *Methods in Molecular Biology*.

[7] Zhou R., Chen K. K., Zhang J. (2018). The decade of exosomal long RNA species: an emerging cancer antagonist. *Molecular Cancer*.

[8] Jarroux J., Morillon A., Pinskaya M. (2017). History, discovery, and classification of lncRNAs. *Advances in Experimental Medicine and Biology*.

[9] Peng Z., Liu C., Wu M. (2018). New insights into long noncoding RNAs and their roles in glioma. *Molecular Cancer*.

[10] Lin C., Yang L. (2018). Long Noncoding RNA in Cancer: Wiring Signaling Circuitry. *Trends in Cell Biology*.

[11] Slaby O., Laga R., Sedlacek O. (2017). Therapeutic targeting of non-coding RNAs in Cancer. *Biochemical Journal*.

[12] Coccia E. M., Cicala C., Charlesworth A. (1992). Regulation and expression of a growth arrest-specific gene (gas5) during growth, differentiation, and development. *Molecular and Cellular Biology*.

[13] Ma C., Shi X., Zhu Q. (2016). The growth arrest-specific transcript 5 (GAS5): a pivotal tumor suppressor long noncoding RNA in human cancers. *Tumor Biology*.

[14] Chang L., Li C., Lan T. (2016). Decreased expression of long non-coding RNA GAS5 indicates a poor prognosis and promotes cell proliferation and invasion in hepatocellular carcinoma by regulating vimentin. *Molecular Medicine Reports*.

[15] Tu Z.-Q., Li R.-J., Mei J.-Z., Li X.-H. (2014). Down-regulation of long non-coding RNA GAS5 is associated with the prognosis of hepatocellular carcinoma. *International Journal of Clinical and Experimental Pathology*.

[16] Hu L., Ye H., Huang G. (2016). Long noncoding RNA GAS5 suppresses the migration and invasion of hepatocellular carcinoma cells via miR-21. *Tumor Biology*.

[17] Yang C., Wu D., Gao L. (2016). Competing endogenous RNA networks in human cancer: hypothesis, validation, and perspectives. *Oncotarget*.

[18] Qu J., Li M., Zhong W., Hu C. (2015). Competing endogenous RNA in cancer: a new pattern of gene expression regulation. *International Journal of Clinical and Experimental Medicine*.

[19] Meng N., Li Y., Zhang H., Sun X. F. (Aug 2008). RECK, a novel matrix metalloproteinase regulator. *Histology and Histopathology*.

[20] Noda M., Oh J., Takahashi R., Kondo S., Kitayama H., Takahashi C. (2003). RECK: A novel suppressor of malignancy linking oncogenic signaling to extracellular matrix remodeling. *Cancer and Metastasis Reviews*.

[21] Wang L., Ge J., Ma T. (2015). Promoter hypermethylation of the cysteine protease RECK may cause metastasis of osteosarcoma. *Tumor Biology*.

[22] Furumoto K., Arii S., Mori A. (2001). RECK gene expression in hepatocellular carcinoma: Correlation with invasion-related clinicopathological factors and its clinical significance. *Hepatology*.

[23] Jia L., Luo S., Ren X. (2017). miR-182 and miR-135b Mediate the Tumorigenesis and Invasiveness of Colorectal Cancer Cells via Targeting ST6GALNAC2 and PI3K/AKT Pathway. *Digestive Diseases and Sciences*.

[24] Zhang Z., Che X., Yang N. (2017). miR-135b-5p Promotes migration, invasion and EMT of pancreatic cancer cells by targeting NR3C2. *Biomedicine & Pharmacotherapy*.

[25] Jin H., Luo S., Wang Y. (2017). miR-135b Stimulates Osteosarcoma Recurrence and Lung Metastasis via Notch and Wnt/*β*-Catenin Signaling. *Molecular Therapy - Nucleic Acids*.

[26] Li Y., Xu D., Bao C. (2015). MicroRNA-135b, a HSF1 target, promotes tumor invasion and metastasis by regulating RECK and EVI5 in hepatocellular carcinoma. *Oncotarget *.

[27] Clark J. C. M., Thomas D. M., Choong P. F. M., Dass C. R. (2007). RECK—A newly discovered inhibitor of metastasis with prognostic significance in multiple forms of cancer. *Cancer and Metastasis Reviews*.

[28] Valeri N., Braconi C., Gasparini P. (2014). MicroRNA-135b promotes cancer progression by acting as a downstream effector of oncogenic pathways in colon cancer. *Cancer Cell*.

[29] Oh J., Takahashi R., Kondo S. (2001). The membrane-anchored MMP inhibitor RECK is a key regulator of extracellular matrix integrity and angiogenesis. *Cell*.

[30] Span P. N., Sweep C. G. J., Manders P., Beex L. V. A. M., Leppert D., Lindberg R. L. P. (2003). Matrix metalloproteinase inhibitor reversion-inducing cysteine-rich protein with Kazal motifs: A prognostic marker for good clinical outcome in human breast carcinoma. *Cancer*.

[31] Alexius-Lindgren M., Andersson E., Lindstedt I., Engström W. (2014). The RECK gene and biological malignancy-its significance in angiogenesis and inhibition of matrix metalloproteinases. *Anticancer Reseach*.

[32] Pramanik K. K., Singh A. K., Alam M. (2016). Reversion-inducing cysteine-rich protein with Kazal motifs and its regulation by glycogen synthase kinase 3 signaling in oral cancer. *Tumor Biology*.

