# Supplementary material for: GAS5 Regulates RECK Expression and Inhibits Invasion Potential of HCC Cells by Sponging miR-135b
Source: Biomed Res Int. 2019 Jan 13;2019:2973289. doi: 10.1155/2019/2973289 (PMC6348854; doi:10.1155/2019/2973289)
Supplement: Supplementary Materials — Supplementary Table I: the clinicopathological characteristics of the donor patients in this study. [file 2973289.f1.pdf]

Supplementary Table I. The clinicopathological characteristics of the donor patients in this study.

No.	Gender	Age	Tumor Size (cm)	Tumor Number	Tumor differentiation	HBV Infection	GAS5 ΔΔCt	miR-135b ΔΔCt
1	Female	53	≥5	Singular	Well	No	0.237	2.333
2	Male	27	≥5	Multiple	Poor	Yes	-2.255	1.756
3	Female	57	≥5	Singular	Moderate	Yes	-0.752	0.825
4	Male	44	<5	Singular	Poor	Yes	1.292	-2.257
5	Male	50	<5	Multiple	Poor	No	-0.35	1.825
6	Male	52	≥5	Singular	Moderate	No	-2.208	-0.449
7	Male	61	≥5	Multiple	Well	Yes	1.795	1.253
8	Female	39	≥5	Singular	Poor	No	0.432	-2.42
9	Male	46	≥5	Singular	Poor	No	-5.556	1.796
10	Female	55	<5	Singular	Well	Yes	-0.209	-1.354
11	Male	52	≥5	Multiple	Poor	No	-3.617	-0.252
12	Female	68	≥5	Singular	Moderate	Yes	0.445	1.834
13	Male	61	<5	Singular	Poor	No	1.076	-1.02
14	Male	67	≥5	Multiple	Moderate	Yes	-4.32	3.894
15	Male	40	<5	Singular	Poor	Yes	-2.54	2.08
16	Female	59	<5	Singular	Poor	Yes	0.508	-0.343
17	Male	45	≥5	Singular	Moderate	No	2.473	-1.19
18	Female	67	≥5	Multiple	Well	Yes	-1.19	2.825
19	Male	40	<5	Singular	Moderate	No	-1.23	1.976
20	Male	62	<5	Multiple	Poor	No	-0.996	1.13
21	Female	58	≥5	Singular	Moderate	Yes	-2.356	1.057
22	Male	66	≥5	Singular	Poor	No	-4.29	3.085
23	Female	48	<5	Multiple	Poor	Yes	0.355	-1.25
24	Male	49	≥5	Multiple	Well	Yes	2.152	-0.927
25	Male	68	≥5	Singular	Well	Yes	1.17	-2.333
26	Female	34	≥5	Singular	Moderate	No	-3.49	1.849
27	Male	42	<5	Singular	Poor	No	-2.576	2.596
28	Female	57	≥5	Singular	Moderate	Yes	-1.032	3.417
29	Male	55	≥5	Multiple	Moderate	Yes	0.977	1.21
30	Male	25	<5	Singular	Poor	Yes	1.25	-0.875
31	Male	51	<5	Singular	Poor	No	-2.35	4.59
32	Male	55	<5	Singular	Moderate	Yes	-0.826	2.832
33	Female	48	≥5	Singular	Poor	Yes	1.796	-1.764
34	Male	39	≥5	Multiple	Poor	Yes	-2.392	3.062
35	Male	52	<5	Multiple	Moderate	No	-0.755	1.078
36	Male	50	<5	Multiple	Well	Yes	-1.38	2.14
37	Female	33	<5	Singular	Poor	No	1.104	0.878
38	Female	62	<5	Multiple	Poor	Yes	2.41	-1.35
39	Male	67	≥5	Singular	Moderate	Yes	-2.076	3.041

**40**	**Male**	**59**	**≥5**	**Singular**	**Poor**	**No**	**-1.199**	**1.692**
**41**	**Male**	**33**	**<5**	**Multiple**	**Poor**	**Yes**	**-0.545**	**0.434**
**42**	**Female**	**43**	**≥5**	**Singular**	**Poor**	**No**	**-3.179**	**1.75**
**43**	**Female**	**46**	**<5**	**Multiple**	**Well**	**Yes**	**-0.458**	**-2.074**
**44**	**Female**	**57**	**<5**	**Singular**	**Poor**	**No**	**0.333**	**-1.112**
**45**	**Male**	**57**	**<5**	**Multiple**	**Moderate**	**No**	**-4.12**	**3.669**
**46**	**Male**	**64**	**≥5**	**Singular**	**Poor**	**No**	**-2.093**	**4.192**
**47**	**Male**	**38**	**<5**	**Multiple**	**Poor**	**Yes**	**-1.17**	**2.37**
**48**	**Female**	**56**	**≥5**	**Multiple**	**Moderate**	**No**	**-0.595**	**-1.32**
**49**	**Male**	**39**	**<5**	**Multiple**	**Moderate**	**Yes**	**0.278**	**-2.042**
**50**	**Female**	**55**	**≥5**	**Singular**	**Poor**	**No**	**-1.005**	**0.843**

ΔΔCt: ΔCt(N)–ΔCt(T). ΔCt(N): Ct value of β-actin or U6B was subtracted from Ct value of GAS5 or miR-135b in normal tissues. ΔCt(T): Ct value of β-actin or U6B was subtracted from Ct value of GAS5 or miR-135b in paired HCC tissues.
